# Three-dimensional reconstructions of Lenke 1A curves

**DOI:** 10.1186/s13013-017-0149-4

**Published:** 2018-02-02

**Authors:** J-C. Bernard, E. Berthonnaud, J. Deceuninck, L. Journoud-Rozand, G. Notin, E. Chaleat-Valayer

**Affiliations:** 1 Croix Rouge française – CMCR des Massues, 92, rue Edmond Locard, 69322 Lyon Cedex 05, France; 2 Hôpital Nord Ouest de Villefranche sur Saône, Gleizé, 69400 France; 3 Laboratoire de Physiologie de l’Exercice, Saint Etienne, France; 4 Etablissements Lecante, Lyon, France

**Keywords:** Idiopathic scoliosis, Thoracic, 3D reconstruction, Upper view, Lenke classification, Regional planes

## Abstract

**Background:**

Scoliosis is a 3D deformity that can be reconstructed through 2D antero-posterior and lateral radiographs, which provide an upper view of the deformed spine as well as regional planes matching all vertebrae of elective plane for each curve. The objective of this study is to explore whether all idiopathic scoliosis classified Lenke 1A have the same 3D representation made with regional planes.

**Methods:**

All patients treated for idiopathic thoracic scoliosis during the growth period and classified Lenke 1A were included in this study conducted in the pediatric spinal orthopedic department of Centre des Massues. A photogrammetric technique was used to obtain a 3D reconstruction, from regional planes identified on radiographs made with the EOS system. Three regional planes are usually identified in asymptomatic spines: lumbar, dorsal, and cervical—none of them presenting rotation. In the studied group, the number of planes, the rotation, and the limit vertebrae of each plane were looked for.

**Results:**

Sixty-three patients were included (47 girls and 16 boys, mean age 11.3 years). The Cobb angle was meanly 36.5°. The scoliosis was reconstructed with three regional planes (57%) or four ones (43%, with the thoracic plane divided into two planes). Maximal rotation was found in the thoracic plane, especially when scoliosis was represented with four regional planes. The transition between planes 2 and 3 was mainly located between the fourth and sixth dorsal vertebrae.

**Conclusion:**

The use of an arbitrary regional plane representation of a 3D shape leads to conclude that there are two types of Lenke 1A scoliosis, which should be taken into account for designing the brace.

## Background

Scoliosis is defined as a three-dimensional deformity in frontal, sagittal, and horizontal planes [1–5]. For Berthonnaud et al., the spine is considered as a heterogeneous beam and is modeled as a deformable wire along which vertebrae can be seen as beads turning on this wire [6]. In our modeling, the 3D spinal curve is a compound of plane regions connected together by zones of transition. The 3D spinal curve is uniquely flexed along the plane regions. Biplanar radiographic examination with simultaneous exposures (frontal and sagittal in the EOS system), coupled with photogrammetric reconstructions, may be used for reconstructing the 3D spinal curve [7]. The photogrammetric technique reconstructs points in space from their two images in projection planes. The photogrammetry applied to radiographic images has been described by Suh [8] and the first presentation of photogrammetric reconstruction of spinal curves from simultaneous biplanar radiography was done by Brown et al. [9]. Biplanar radiography involves the setting of specific devices to get simultaneous exposures, and this has been used for clinical applications. The geometric structure of a 3D spinal curve can be characterized by the size and orientation of regional planes, by the parameters representing flexed regions and by the size and function of zones of transition [6].

Despite the fact that all classifications are only based on 2D like the Scoliosis Research Society (SRS) classification [10] and King classification [11, 12], Lenke introduces with his classification new parameters in radiographic analysis of idiopathic scoliosis, such as lumbar sagittal modifiers (A, B, C) and the difference between structural and non-structural curves [13, 14].

The objective of this study was to show if the use of regional plane analysis could determine if all Lenke 1A curves (main thoracic = thoracic scoliosis without compensation on the lumbar part) would result in the same 3D representation.

## Methods

To become familiar with Lenke classification, we classified all the radiographs of patients who consulted for adolescent idiopathic scoliosis and underwent frontal and sagittal radiographs on the EOS system in Centre des Massues in 2015. Although the Lenke classification has already proved its reliability [13], four independent readers (two very familiar with scoliosis and two not) analyzed 223 files, and then we compared results. In order to distinguish Lenke type 1 from type 2, we did not use bending radiographs to determine whether the upper curve was structural or not, because it is difficult in daily practice to multiply radiographs for these patients in the growth period, as they are already very often exposed. We used clinical examination for that, by measuring the upper bump in standing position and comparing it to the one measured in ventral decubitus: if the upper bump disappeared in lying position, it means that the curve was not structural. When we all agreed that the scoliosis could be classified as Lenke 1A, we kept the case and included it in our group for this study.

All patients with Lenke 1A curves who consulted in our institution and underwent frontal and sagittal radiographs on the EOS system in 2015 were recruited. Patient’s characteristics were recorded: age, height, weight, and radiographic measures: Cobb angles, pelvic incidence (PI), pelvic tilt (PT).

**Fig. 1:**
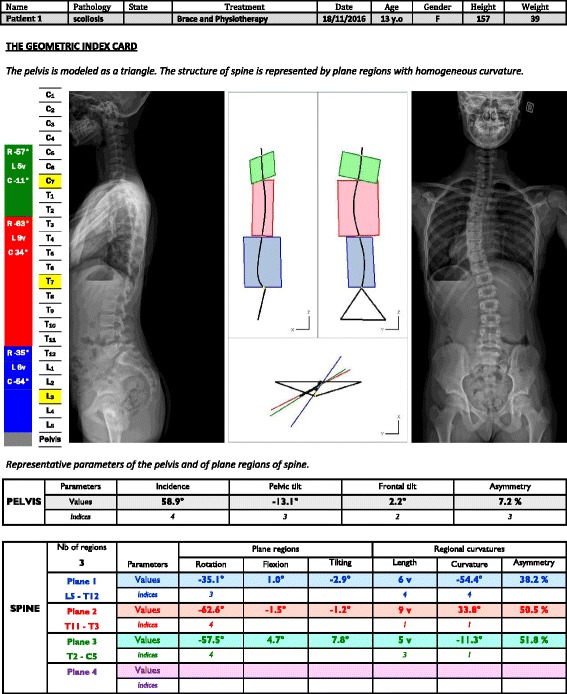
Example of 3D reconstruction of Lenke 1A scoliosis with three regional planes identified on the lateral, frontal, and horizontal views: the blue plane is for the lumbar region, the red plane for the dorsal region, and the green plane for the cervico-thoracic region

**Fig. 2:**
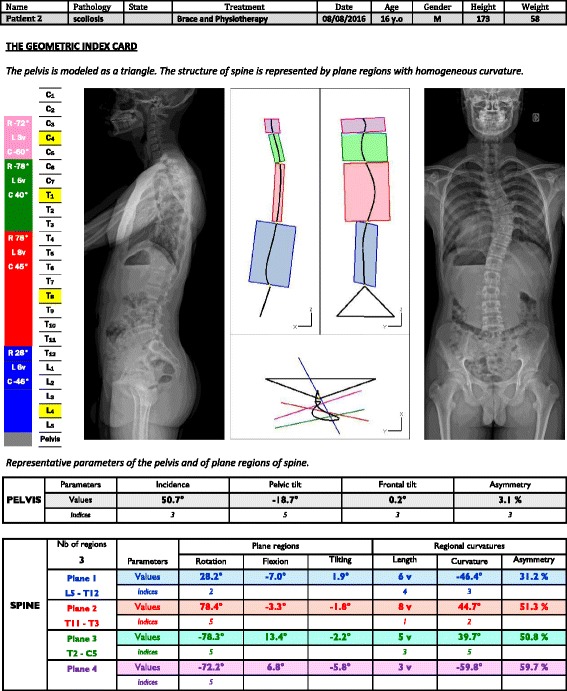
Example of 3D reconstruction of Lenke 1A scoliosis with four planes identified on the lateral, frontal, and horizontal views: the blue plane is for the lumbar region, the red plane for the lower dorsal region, the green plane for the cervico-thoracic region, and the pink plane for the upper cervical region

Median spinal curves were drawn on frontal and sagittal projections. Points of frontal and sagittal curves were then linked together for the photogrammetric reconstruction of the 3D spinal curve. The relation was based on the use of epipolar planes. The 3D spinal curves were projected on fixed plane, and regional planes were detected along this rough spinal curve [6, 7]. Three or four planes were then identified. Figure 1 shows an example of 3D reconstruction of Lenke 1A scoliosis with three planes, whereas Fig. 2 shows an example with four planes.

### Statistical analysis

All of the collected information was coded and subsequently captured by computer equipment using SPSS 11.5 software for analysis, which is carried out with the support of the suitable statistical tests (ANOVA and correlation study). The pelvic and spinal parameters have been chosen as dependent variables. Correlation analysis between radiological data and 3D data was performed through the use of Spearman’s rank correlation coefficient.

Comparison of the mean values for each of the pelvic and spinal parameters between the groups was carried out through non-parametric analysis of variance (ANOVA). For all the tests, the degree of statistical significance was set at *P* < 0.05.

## Results

A total of 63 Lenke 1A patients were included (mean age 11.3 years, with 47 girls and 16 boys). The thoracic Cobb angle in the frontal plane ranged between 14° and 70° (mean 36.5°).

Patient characteristics are presented in Table 1.

**Table 1:** Demographic characteristics and radiographic measures for pelvic incidence (PI) and pelvic tilt (PT)

*n* = 63				
	Mean	SD	Min	Max
Age	11.3 (year old)	2.6	7	15
Weight	47.3 (kg)	10.9	20	70
Height	157.4 (cm)	12.1	120	179
PI	50.9 (degree)	9.7	26	78
PT	9.3 (degree)	7.4	−6	29

Table 2 presents the data for the three planes in our sample. The thoracic plane was the most rotated, but we found also rotation in the lumbar plane and in the upper plane as well.

**Table 2:** Horizontal rotation of the 3 planes

		Mean	SD	Min	Max
Rot 1	Rotation of the lumbar plane	33.6 (degree)	18.8	−15.7	67.5
Rot 2	Rotation of the thoracic plane	45 (degree)	55.6	−88.8	89
Rot 3	Rotation of the cervical plane	−23.5 (degree)	51.2	−86.7	85.7

**Fig. 3:**
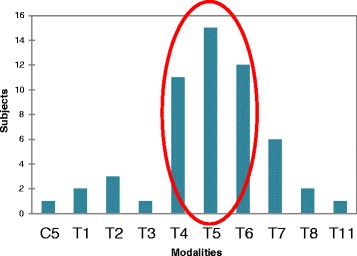
Location of the junction between the second and the third plane in our sample of Lenke 1A scoliosis

In our population of Lenke 1A scoliotic patients, the rotation was maximal in the second plane which represents the thoracic plane. The transition from the thoracic plane to the upper plane (between plane 2 and plane 3) occurred mainly between the fourth and the sixth thoracic vertebrae, as shown by Fig. 3.

**Fig. 4:**
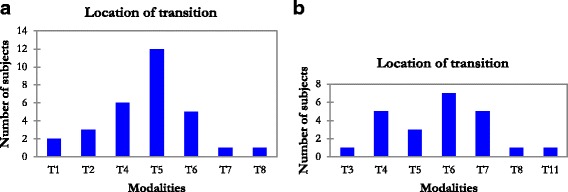
Location of the junction between the second and the third plane in our sample of Lenke 1A scoliosis, divided into two groups: the three-plane group (**a**) and the four-plane group (**b**)

In 62.3% of cases, the rotation of the third plane was negative (clockwise direction) and positive in 37.7% (counterclockwise direction). We found no correlation between the Cobb angle in the frontal plane and the Cobb angle in the regional plane (*p* = 0.298). We identified three regional planes in 57% of cases and four regional planes in 43% of cases (when the thoracic plane was divided into two parts), and we found a difference between the three-plane group and the four-plane group for the rotation of the thoracic plane, as shown by Table 3 and Fig. 4. This difference was statically not significant, but plane 2 tends to rotate more when the 3D reconstruction identified four planes than when the 3D reconstruction identified three planes.

**Table 3:** Pelvic incidence (PI), pelvic tilt (PT), and horizontal rotation of the first three planes for both groups, identified either with three or four planes

	*n* = 63				
	*n* = 36 (57%)		*n* = 27 (43%)		
	3 planes		4 planes		*p* value
	Mean	SD	Mean	SD	
PI	52.4	9.6	49	9.8	0.209
PT	8.3	7.8	10.5	6.9	0.293
Rot 1	31.9	18.2	35.8	19.7	0.451
Rot 2	67.6	29.4	77.2	16.4	*0.167*
Rot 3	−22.7	52.8	−24.6	50.1	0.89

In the three-plane group, the mean rotation for the thoracic plane (Rot 2) was 67.6° ±29.4 and the transition from plane 2 to plane 3 was mainly located in T5. We found a correlation between the rotation of the plane and the level of transition (*p* < 0.0001).

In the four-plane group, the mean rotation for the thoracic plane (Rot 2) was 77.2° ±16.4 and the transition from plane 2 to plane 3 was mainly located in T6/T7. There was also a correlation between the rotation of the plane and the level of transition (*p* = 0.005).

## Discussion

The aim of this work was to study the Lenke 1A thoracic idiopathic scoliosis in 3D using the specific software Optispine® [15] in order to determine if all Lenke 1A curves are similar in 3D analysis.

In daily practice, classifications such as SRS [10], Lenke [13, 14], and King [11, 12] are the most common for the surgical or orthopedic treatment of scoliosis. These classifications are based on 2D radiographs of the spine in a standing position: postero-anterior view for SRS and antero-posterior and profile views for Lenke.

The Cobb angle helps in defining the severity of scoliosis by measuring curves in frontal and sagittal planes. The emergence of 3D analysis to evaluate scoliosis [6, 7, 13, 16–21] has allowed a more comprehensive and more correct approach, thanks to the top view.

Among all parameters that have been defined to describe 3D deformation in scoliosis, we retain the plane of major curvature (PMC), the best-fit planes, and the regional planes [6, 14, 22]. This study was conducted with the regional planes, and our results show that there is no correlation between the Cobb angle simply measured on a front radiograph in standing position and the angle measured on the matching regional plane. This measure on regional plane is done semi-automatically by considering as limits some vertebrae that might not have been identified as such on a frontal radiograph. The Cobb angle on a regional plane is always higher than or equal to the Cobb angle measured on a frontal radiograph. This observation is consistent with the work of Duong and Mac-Thiong [22] who have previously shown how complex it is to classify scoliosis in 3D, specifically for Lenke 1 curves. The earlier works of Stagnara [16, 23] are also in line with our results. He used to measure scoliosis with the Cobb angle on an antero-posterior plane and completed his radiographic assessment for high-range scoliosis with a specific view, characterized by an incidence called “elective plane” on the main curve [24]: the spinal deformity is major when projected on a perpendicular plane to this specific incidence. The curve is then measured by the Cobb method and the range of deformity is compared to the one measured in standard conditions, as previously described. The Cobb angle measured on the elective plane (strict front view of apical vertebrae) was always higher than the Cobb angle measured on the front radiograph.

In asymptomatic subjects, the spinal column is made of three regional planes and three junction points [15]: plane 1 for lumbar level, plane 2 for dorsal level, and plane 3 for cervical level. The results of our study show that in scoliotic patients, plane 3 includes dorsal vertebrae from T4, T5, or T6 in 40% of cases, whereas it includes other dorsal levels in 60% of cases.

Compared to the best fit plane (BFP) with fix limit vertebrae, as described by Duong et al. [22], 3D analysis of scoliosis through regional planes highlights different levels of junction point between plane 2 (thoracic plane) and plane 3, even if the same kind of Lenke curve is considered (i.e., main thoracic). The more rotated the thoracic plane, the lower the junction at the thoracic level. It seems thus interesting to analyze the direction of rotation and to identify the vertebral “breaking point” of this plane 3. Regarding plane rotation, this study reveals that plane 1 (i.e., lumbar plane) mainly presents a direction of rotation similar to the dorsal plane (positive). Plane 1 always rotates less than plane 2, and plane 3 also rotates less than plane 2. Let us specify here again that this work focuses on Lenke 1A scoliosis, which may explain that the rotation of plane 1 is moderate: it would probably have been different if we had considered Lenke 1B or 1C scoliosis, in which thoracic scoliosis is associated with a lumbar non-structural curve destabilized in relation to the center of the sacral plate.

By distinguishing Lenke 1A scoliosis made of three planes from Lenke 1A scoliosis made of four planes after 3D reconstruction, we were able to observe that plane 2 (i.e., thoracic plane) in four-plane scoliosis shows a more important rotation than in three-plane scoliosis. We can thus suppose that the importance of deformity may be responsible for the plane break. Similarly, the junction between plane 2 and plane 3 in four-plane scoliosis is lower when the deformity is more important. In practice, for orthopedic conservative treatment, the first objective with the brace will be to reduce plane rotations, which will lead to a reduction of the Cobb angles and if possible guide this scoliosis from four planes to three planes.

This work also brings to light the fact that the rotation of the cervico-thoracic plane (i.e., plane 3) may either be positive (in counterclockwise direction) or negative (in clockwise direction). Our results show that similar thoracic scoliosis, from a clinical and standard radiographic point of view, may be different from a 3D perspective, with plane 3 presenting either a positive rotation (in the same direction as the dorsal plane) or a negative one (opposite to plane 2)—even if this study does not allow us to come up with an explanation.

We note that Lenke 1A scoliosis could be divided into two classes, depending on the direction of rotation of plane 3 (positive or negative).

The heterogeneity of Lenke 1A has already been demonstrated by Atmaca [25], who added an axial plane analysis to conventional coronal and sagittal evaluations and concluded that it could reveal inherent structural differences that are not apparent in single planar radiographic assessments and may necessitate a different surgical strategy. We also think that this analysis is important to orient treatments, and are convinced that the radiographic evaluation of scoliosis should not be only descriptive in 2D but should also wherever possible be completed by a 3D analysis (upper view), taking into account the regional planes. In daily practice, our evaluation has to consider the Cobb angle on the regional plane and the rotation of the plane. For all that, this analysis is based on a wired reconstruction, which is a limitation for the use of this software, as it does not provide a view of the ribcage (which is important for the conception of the brace) [26]. 3D analysis does not exempt us from a thorough reading of 2D front and profile pictures, in order to have a good radiographic and medical interpretation of issues regarding the spine, ribs, and soft tissues.

## Conclusion

All scoliotic curves classified 1A from 2D radiographs do not have the same 3D representation. This work underlines the importance of using 3D reconstructions to analyze scoliosis, that is to say including a front view, a profile view, and also a horizontal or top view, in order to assess precisely and understand the deformity. This kind of analysis is particularly useful to identify how scoliosis evolves, by measuring the Cobb angle in regional planes and by quantifying the rotation of the plane, all the while comparing these parameters to clinical data.

Nowadays, the evaluation of scoliotic deformity is based on clinical analysis and morphometric assessment of the trunk, completed by radiographic examination. One needs to go further and use these data to obtain a top view of scoliosis and regional planes, especially when therapeutic options are discussed. To be still more precise, all studied parameters should be connected to their impact on muscular function, and vice versa.

This work can be the starting point for further studies to investigate thoracic scoliosis, depending on the Cobb angle on the plane, number of consecutive planes, and location of breaking point between plane 2 and plane 3, as well as direction of rotation of plane 3. And it will possibly help to develop more adapted therapeutic strategies, especially when considering brace treatment. This has completely changed our daily clinical practice, with a real concern about the orientation and the importance of forces that should be applied through the brace’s pads. The top view allows a representation of scoliosis closer to reality and helps to define the ideal brace to correct it.

This regional planes analysis considers each curvature with its rotation, and this is the importance of rotation that helps us to decide and prioritize the to-be-treated curvatures. This is also very helpful to understand why some scoliosis remain stable after brace removal (because the planes rotation is well corrected) whereas others can still become worth, as the plane rotation is not corrected, even if the Cobb angle is corrected on frontal radiographs.

## Abbreviations

2D: Two dimensional
3D: Three dimensional
BFP: Best fit plane
PI: Pelvic incidence
PMC: Plane of major curvature
PT: Pelvic tilt
SRS: Scoliosis Research Society
